# Enhancing autophagy and energy metabolism in the meniscus can delay the occurrence of PTOA in ACLT rat

**DOI:** 10.3389/fcell.2022.971736

**Published:** 2022-09-02

**Authors:** Huangrong Zhu, Hai Liu, Xizhong Chen, Xin Xu, Shuqin Zhang, Denghui Xie

**Affiliations:** ^1^ The Fourth Affiliated Hospital, Zhejiang University School of Medicine, Yiwu, China; ^2^ Department of Joint Surgery, Center for Orthopaedic Surgery, The Third Affiliated Hospital of Southern Medical University (Academy of Orthopedics), Guangzhou, China; ^3^ Orthopedic Hospital of Guangdong, Guangzhou, China; ^4^ Guangdong Provincial Key Laboratory of Bone and Joint Degeneration Diseases, The Third Affiliated Hospital of Southern Medical University, Guangzhou, China

**Keywords:** PTOA, ACLT, energy metabolism, autophagy regulation, meniscus, articular cartilage

## Abstract

Osteoarthritis (OA) is a progressive degenerative joint disease characterized by the destruction of the articular cartilage, meniscus and the like. Autophagy and cellular energy metabolism are the mechanisms by which cells maintain homeostasis. However, little is known about the effects of autophagy and cellular energy metabolism on meniscus degeneration, and the pathogenesis of posttraumatic osteoarthritis (PTOA) after the meniscal injury is rarely reported. Therefore, this study aimed to investigate the relationship between changes in autophagy and cellular energy metabolism in the meniscus following anterior cruciate ligament transection (ACLT) and PTOA induced by subsequent articular cartilage injury. In this study, we use a combination of cell experiments *in vitro* and animal experiments *in vivo*. On the one hand, cell experiment results show that inhibiting the mTORC1 signaling pathway by inhibiting the phosphorylation of S6K and AKT proteins in meniscal cells will lead to the increase of Beclin1, LC-3B, ATG12, ULK1, P62, and activate autophagy-related signaling pathways, which in turn protects the extracellular matrix component COL1 of meniscal cells from degradation by catabolic factor MMP13. In addition, it increased the generation of mitochondrial membrane potential in meniscal cells, increased the expression of anti-apoptotic factor BCL-XL, decreased the expression of pro-apoptotic factors BAD and BAX, and reduced the apoptosis of meniscal cells. More importantly, under the stimulation of inflammatory factor IL-1β, the secretion of meniscus cells can reduce the elevated levels of MMP13 and Adamts5 caused by chondrocytes affected by IL-1β. On the other hand, the results of animal experiments *in vivo* further proved the validity of the results of the cell experiments, and also proved that the meniscus injury did prior to the articular cartilage degeneration after ACLT. In conclusion, this study suggests that the meniscus prior to articular cartilage damage during the development of PTOA after ACLT, and that promoting autophagy and energy metabolism of meniscal cells may be a potential therapeutic target for delaying PTOA.

## Introduction

OA is a common geriatric disease that mainly causes joint pain and dysfunction, and brings a huge burden to the family and society (Koushesh et al., 2022). OA is traditionally characterized by cartilage erosion and subchondral bone sclerosis (Housmans et al., 2022). It has now been established that OA is a progressive degenerative disease that endangers the structure of the joint, which is characterized by the destruction of all structures in the joint, including subchondral bone, meniscus, and synovium and other tissues (Martel-Pelletier et al., 2016).

The exact mechanism of the occurrence and development of OA is unclear, but it mainly affects weight-bearing joints, the hip and knee joints. Age, gender, genetic susceptibility and obesity are the main risk factors for the development of OA (Mandl, 2019). In recent years, the high incidence of OA and the huge medical and social costs have motivated researchers to study in this field (Butterfield et al., 2021). It has now been known that inflammation is the main cause that primarily affects the degeneration of articular cartilage, and although the level of inflammation in osteoarthritis is much lower compared to inflammatory arthritis, inflammation in osteoarthritis is present in different joint tissues, such as cartilage, subchondral bone, and synovium. Thus, osteoarthritis is due to an imbalance of catabolism and anabolism of articular cartilage and the development of secondary compensatory changes that ultimately lead to dysfunction of the entire joint (Robinson et al., 2016; Griffin and Scanzello, 2019). The main factors affecting the catabolic process in OA are interleukin-1β (IL-1β) and tumor necrosis factor-α (TNF-α). OA destroys the extracellular matrix and chondrocytes in articular cartilage, the matrix mainly containing collagen II, collagen IX and collagen XI, and proteoglycans (Wang and He, 2018). A constant feature in OA has increased the production of cartilage-degrading enzymes, including matrix metalloproteinases (MMPs), stromelysins (MMP-3), gelatinases (MMP-9), matrixes (MMP-7), and collagenases13 (MMP-13) and Adamts (Malemud, 2019). In addition to inflammatory factors, increased expression of transforming growth factor-β (TGF-β) and insulin-like growth factor (IGF) was found in the cartilage of OA patients and positively correlated with the degree of cartilage destruction. However, no increase in TGF-β and IGF levels was observed in healthy subjects. Furthermore, increased expression of TGF-β and IGF was detected in osteophytes, and repeated injections of TGF-β and IGF resulted in osteophyte formation in an animal model of OA (MacFarlane et al., 2017; Wen et al., 2021). Further research found that subchondral bone plays a key role in the pathogenesis of OA. Subchondral bone is located below calcified cartilage and consists of the subchondral bone plate and subchondral trabecular bone. The subchondral bone plate is a thin layer of cortex adjacent to the calcified cartilage and provides the connection between the articular cartilage and the subchondral trabecular bone. The role of the subchondral bone is thought to be to support the articular cartilage above and to distribute mechanical loads acting on the articular surface. Therefore, stiffness of the subchondral bone may transmit an increased load to the articular cartilage causing damage. Hence, it is considered that subchondral bone also plays an important role in OA, and subchondral sclerosis caused by thickening of the subchondral bone plate and increased trabecular thickness is a recognized imaging manifestation, especially in the late stage of OA (Osterberg et al., 2017; Hu et al., 2021).

Studies have shown that TOR, the target of rapamycin, acts as a central hub in a highly conserved signaling network that controls various aspects of cellular and organismal physiology. Thus, dysregulation of mTOR signaling in humans is associated with a variety of diseases, including metabolic syndrome, cancer, and neurodegenerative diseases. TOR assembles into two distinct complexes: rapamycin-sensitive TORC1 and rapamycin-insensitive TORC2, with distinct subunit compositions and downstream targets, respectively (Wullschleger et al., 2006). Relative to mTORC2, rapamycin-sensitive mTORC1 signaling is thus far easier to study, and many of its downstream targets and upstream regulatory mechanisms have been identified. When activated, mTORC1 initiates a phosphorylation cascade involving multiple substrates that work together to promote cell growth and inhibit autophagy. This growth-promoting mechanism includes the upregulation of protein synthesis, nucleotide synthesis, and lipid synthesis as well as aerobic glycolysis. Since mTORC1 acts as a central hub in numerous signal transduction mechanisms, it is tightly controlled (Ben-Sahra and Manning, 2017). Autophagy was primarily discovered when looking at the structure of rat liver lysosomes. Although the importance of autophagy in mammalian organisms is well recognized, how autophagy regulates body functions remains unclear. Autophagy is highly active in most cell types and presumably plays a housekeeping role in maintaining the integrity of intracellular organelles and proteins. However, autophagy is induced by starvation and is a key component of the adaptive response of cells and organisms to nutrient deprivation, so nutrient deficiencies promote autophagy. Mainly involved in nutrient sensing, regulation of cell growth and autophagy is the rapamycin (TOR) kinase, which is downstream-regulated by growth factor receptor signaling, hypoxia, ATP content and insulin signaling. TOR kinase increases protein translation and inhibits autophagy through a series of instructions when nutrients are abundant (Rabanal-Ruiz et al., 2017; Liu and Sabatini, 2020).

Energy metabolism, also known as central carbon metabolism (CCM), maintains the most basic activities of life, usually including glycolysis (EMP), tricarboxylic acid cycle (TCA) and pentose phosphate pathways (PPP), It also includes the decomposition of amino acids, lipids and other substances to generate substrates and enter the TCA to participate in the process of metabolism. On the one hand, energy metabolism provides energy substances such as ATP for life activities. On the other hand, energy metabolism also provides abundant substrates for the biosynthesis of other amino acids, glucose, nucleic acids and fatty acids (Judge and Dodd, 2020). Oxygen is the final acceptor of oxidative phosphorylation electrons. Under normoxia, aerobic metabolism is the basis of mammalian cell metabolism. Under hypoxic conditions, the utilization of oxygen is limited, the activity of the mitochondrial electron transport chain is reduced, and the energy metabolism pathway is converted from oxidative phosphorylation to glycolysis. Hypoxic also activates the expression of HIFs, upregulates the expression of glycolytic enzymes and glucose transport, and downregulates the expression of mitochondrial enzymes. The cellular energy state sensor, adenosine phosphate-activated protein kinase (AMPK), plays an important role in metabolism. Studies have shown that AMPK can inhibit mTORC1, resulting in a decrease in the rate of cellular oxygen consumption and intracellular ROS, and ultimately affect the life activities of cells (Lunt and Vander Heiden, 2011; Lee et al., 2020).

Research in the last century from total meniscectomy to partial meniscectomy to meniscus transplantation are milestones in understanding the anatomical and functional uses of the knee meniscus and have led to numerous studies of different treatments. The current prevalent treatment for the repair of meniscal injuries is to keep the tissue intact as much as possible. However, in the case of complex or fully traumatic lesions, the surgeon cannot fully restore the meniscus tissue anatomically and functionally, so complete repair after meniscal injury remains a great challenge. At the same time, it also provides a greater impetus to find new treatment options for delaying the progression of PTOA (Makris et al., 2011). Numerous *in vivo* and *in vitro* studies have shown that mechanical factors play an important role in the degeneration of the meniscus, but the process of mechanosignaling involves complex transitions of mechanical and biochemical events, and new insights into the mechanisms of biophysical signaling will hold promise create new physical or drug therapies to prevent the degeneration of the meniscus and enhance the repair of damaged meniscus, thereby delaying the development of PTOA (Rodeo et al., 2020).

In this study, we hypothesized that the meniscus plays a key role in the progression of PTOA, and explored whether the meniscus prior to articular cartilage degeneration in the development of PTOA after ACLT, whether the relationship between them occurs not only through mechano-mechanical conduction, but more likely through molecular biological interaction. To verify whether reversal of meniscal tissue damage after ACLT can delay or even reverse the occurrence of PTOA. Therefore, this study aimed to investigate the relationship between abnormal changes in the meniscus caused by abnormal mechanosignal transduction after ACLT and PTOA caused by subsequent articular cartilage injury.

## Materials and methods

### Materials

The DMEM/F12 used in this experiment was purchased from Corning (United States), the Australian fetal bovine serum was come from Gibco (United States), the TRizol reagent was obtained from Invitrogen (Thermo Fisher Scientific), and the Evo M-MLV Reverse Transcription Kit ((for qPCR) AG11707) and SYBR^®^ Green Pro Taq HS Master Mixed qPCR Kit (AG11701) were shop with Accurate Biotechnology (Hunan)Co., Ltd. the PCR primer sequences were gained from Sangon Biotech (Shanghai)Co., Ltd. the mitochondrial membrane potential detection kit was trade with MedChemExpress (MCE,United States), and the ATP content detection kit was shop at Solarbio Technology (Beijing)Co., Ltd. HE staining solution, Toluidine blue staining powder and safranin O/fast green staining powder was buy from Sigma (United States), SDS-PAGE gel rapid preparation kit was attained from Beyotime Biotechnology (Shanghai)Co., Ltd. rapamycin was acquired from Shanghai yuanye Bio-Technology Co., Ltd., IL-1β was purchased from R&D Systems (Bio-Techne), 3-methyladenine (3-MA, autophagy inhibitor) was produced from TargetMol (United States), primary antibodies MMP13 (18165-1-AP), Adamts5 (Ag26730), P-AKT (Phospho-AKT1 (Ser473)), Beclin 1 (11306-1-AP), LC-3B (18725-1-AP), ULK1 (20986-1-AP), ATG12 (11264-1-AP), COL1 (66761-1-lg) achieved from Proteintech Group, COL2 (GB11021) were procured from Servicebio (China), P-S6K (9208S)/S6K (9202S) were come from Cell Signaling Technology (CST), P62 (ab109012) was purchased from Abcam (Shanghai). Sprague Dawley (SD) rats were purchased from Guangdong Animal Experiment Center.

### The meniscal cell culture and treatment

Meniscal tissue was isolated from rat knee joints under sterile conditions. By digesting with pronase 2 mg/ml in serum-free DMEM/F-12 for 1 h at 37°C, followed by collagenase-P 0.25 mg/ml in DMEM/F-12 with 5% fetal bovine serum Digest overnight. The viability of detached cells was determined using trypan blue and cells were counted using a hemocytometer. Monolayer cultures were established by plating cells in six-well plates at 2 × 106 cells/ml in DMEM/F-12 medium supplemented with 10% fetal bovine serum. The cells were cultured for about 3–5 days, and the medium was changed every 2 days until they reached 100% confluence before the experiment was used. After the cells became confluent, different drug treatments were added according to different experimental groups: in the control group (Control) Add 1ul of PBS per ml of medium, IL-1β group (IL-1β) with 1 ul of 10 ug/ml of IL-1β per ml of medium, IL-1β+rapamycin group (IL-1β+Rapa) was added with 1ul of 10 ug/ml of IL-1β and 100 mM of rapamycin per ml of medium.

### Western blot

The proteins of each group were collected after 24 h of the different interventions. Cellular proteins were separated by SDS polyacrylamide gel electrophoresis and transferred to nitrocellulose membranes (Millipore). Membranes were blocked with 5% (wt/vol) nonfat milk diluted in TBST for 1 h at room temperature. Membranes were then incubated with the corresponding primary antibodies overnight at 4°C. The primary antibody was then incubated with the corresponding secondary antibody conjugate for 1 h at room temperature. The signal was visualized using an enhanced chemiluminescence kit. Western blot data were evaluated as follows: In the control and experimental groups, the gray values of the Western blot bands were measured by Image-ProPlus 4.5 software. Normalize by dividing the gray value of each set of proteins of interest by the gray value of the reference protein.

### RT-qPCR

After 24 h of different interventions, the supernatant in the six-well plate was removed, washed with PBS and then 1 ml of Trizol reagent was added to each well to digest for 5 min according to the instructions, and the mixture was pipetted into a 1.5 ml non-enzyme EP tube. Put 200 ul of chloroform into a 1.5 ml non-enzyme EP tube, shake the EP tube vigorously on a shaker, stand at room temperature for 15 min, and centrifuge at 12,000 rpm at 4°C for 15 min. After centrifugation, it can be seen that the EP tube is divided into three layers. Sucked the upper water phase in the EP tube as much as possible, and moved it to a new EP tube. Subsequently, add 500 ul of isopropanol with a pipette, and shake it fully on the shaker. Stand at room temperature for 10 min, and centrifuge at 12,000 rpm at 4°C for 15 min. After centrifugation, the white precipitate can be seen at the bottom of the tube, carefully remove the supernatant with a pipette, and add 750 ul of 80% alcohol to wash the precipitate up, centrifuge at 10,000 rpm at 4°C for 10 min. After the centrifugation, remove the supernatant with a pipette, leave the precipitate, and place it in a ventilated place to air dry. Add 20 ul of RNase-free DEPC water to the tube with a pipette and dissolve it fully Precipitate. Take 2 ul to measure the RNA concentration on a UV spectrophotometer. According to the operating instructions provided by the company, the extracted RNA was reverse transcribed into cDNA, and real-time quantitative PCR was performed to detect the expression of the target gene in each sample. The specific primer sequences are shown in Supplementary Table S1. The qPCR data were finally analyzed using the 2-ΔΔCt method.

### Mitochondrial membrane potential detection

After 24 h of different interventions, the dye solution was prepared in advance according to the product instructions provided by the company. Remove the supernatant from the six-well plate, add 1 ml of cell culture medium to each well after washing with PBS, take JC-1 (200 μM) to room temperature, and add 10 μl of JC-1 solution to each well of culture medium. Its final concentration is 2 μM. After mixing gently, incubate at 37°C for 15–20 min. Set the positive control group, take the CCCP solution (50 mM) to room temperature, add 1 μl to the culture medium of the positive control wells to a final concentration of 50 μM, and incubate at 37°C for 5 min after mixing gently. After incubation at 37°C, the supernatant was removed and washed twice with PBS (1×). 500 μl of PBS (1×) was added, and the slides were observed under a fluorescence microscope.

### ATP content detection

After 24 h of different interventions, the cells were first collected into a centrifuge tube, the supernatant was discarded after centrifugation, the extract solution was added according to the number of cells, the cells were disrupted with ultrasonic waves for 2 min, and centrifuged at 10,000 rpm at 4°C for 10 min. Aspirate the supernatant into another EP tube, add 500 μl of chloroform, shake and mix on a shaker, centrifuge at 10,000 rpm at 4 °C for 3 min, aspirate the supernatant, and put it on ice for detection. Turn on the UV spectrophotometer and preheat for more than 30 min, adjust the wavelength to 340 nm, and normalization with distilled water. Corresponding sample determination and calculation of ATP content were performed according to the sample instructions.

### Animal experiment

The animal experiments designed by the institute have been reviewed by the Ethics Committee of Animal Experiments of Southern Medical University. Twenty-four Sprague Dawley (SD) male rats aged 12 weeks were randomly divided into 4 groups. The Sham group was the rats whose joint capsule was only opened without ACLT. The right hind limbs of SD rats in the other groups were shaved and washed with povidone-iodine. The specific surgical procedure of ACLT surgery was established according to previous research (Hayami et al., 2012; Lorenz and Grassel, 2014). The detailed surgical procedure is presented in Supplementary Materials (Supplementary Figure S1). Intra-articular injection of different drugs was performed according to the grouping situation. Sham group (Sham): intra-articular injection of 0.02% DMSO solution 200 ul, control group (Control): intra-articular injection of 0.02% DMSO solution 200ul, rapamycin group (Rapa): intra-articular injection of 10uM rapamycin solution 200ul, rapamycin+3 MA group (Rapa+3-MA): intra-articular injection of 10uM rapamycin solution+75 mg/ml 3-MA solution 200ul. According to the experimental design, the intra-articular injection was performed twice a week for one month after ACLT, and the material was collected after continuous medication for 8 weeks. The rats to be sampled were anesthetized with sodium pentobarbital and then sacrificed by decapitation. Use scissors to separate the skin of the rat, cut off the muscles around the joint, cut the femur and tibia, turn the rat’s knee joint to 120°, place it in a centrifuge tube, and add 10 times the volume of 4% paraformaldehyde to fix the specimen. It was left to stand for 48 h and then decalcified, dehydrated, embedded and sliced for subsequent processing.

### Histological staining

HE staining and toluidine blue staining were carried out concerning previous literature reports (Liu et al., 2021; Yan et al., 2021). In short, for HE staining, select paraffin sections with clear joint structure, then place them in a 65°C oven for 1 h and then perform a series of dewaxing and hydration steps. And then nuclei were stained with hematoxylin for 2 min, the excess hematoxylin staining solution was rinsed with running water, differentiated with the prepared differentiation solution for 5 s, PBS incubated for 10 s, stained with eosin for 5 s, rinsed, and then mounted for observation and filming. The steps of section selection, baking, dewaxing and hydration for toluidine blue staining and safranin O/fast green staining are the same as those for HE staining. The sections after dewaxing and hydration are cleaned and then soaked with 0.1% toluidine blue staining solution for 10 min, and the excess dye solution is washed away. The section can be observed after the cover step. For safranin O/fast green staining, the sections were stained with Safranin O staining solution for 5 min, and then stained with Fast Green staining solution for 3 min after washing away the excess dye, and washed again for observation.

### Immunohistochemical staining and immunofluorescence staining

Immunohistochemical staining and immunofluorescence staining were carried out regarding previous literature reports (Liu et al., 2021; Suo et al., 2022). The steps of section selection, baking, dewaxing and hydration are the same as those of HE staining. After dewaxing and hydration, microwave antigen retrieval is performed to better expose intracellular or extracellular antigens. We use the microwave repair method. The tissue slices are placed in a microwave oven at 100°C for 1 min and 30 s until there are more air bubbles (30%–40%) on the slices. The sections were then placed at room temperature to cool down, rinsed with PBS, circled with a special pen for histochemistry, incubated with hydrogen peroxide for 10 min, rinsed with PBS and blocked with goat serum for 1 h, and then incubated with the corresponding primary antibody overnight at 4°C. The next day, the slices were taken out and equilibrated to room temperature, rinsed with PBS, and incubated with the corresponding secondary antibody for 1 h. After rinsing with PBS, DAB developed color and hematoxylin stained nuclei. The section can be observed after the sealing step. Immunofluorescence staining is similar to the above steps, the main difference is that there is no need to drop hydrogen peroxide. It should be noted that after adding the secondary antibody, it should be protected from light, and DyLight (TM) 488 or Alexa Fluor 647 should be used to replace the secondary antibody for labeling After staining, DAPI was added dropwise to stain the nucleus, and pictures were taken by fluorescence microscope.

### Statistical analysis

The data in the article were analyzed by one-way ANOVA, and quantitative data were expressed as mean ± standard deviation. SPSS 26.0 software was used for statistical significance analysis of data, and Graph Pad software was used for graph visualization, *n* = 4–6, *p < 0.05, **p < 0.01, ***p < 0.001.

## Result

### The progression of osteoarthritis was delayed by rapamycin in anterior cruciate ligament transection rats

**FIGURE 1:**
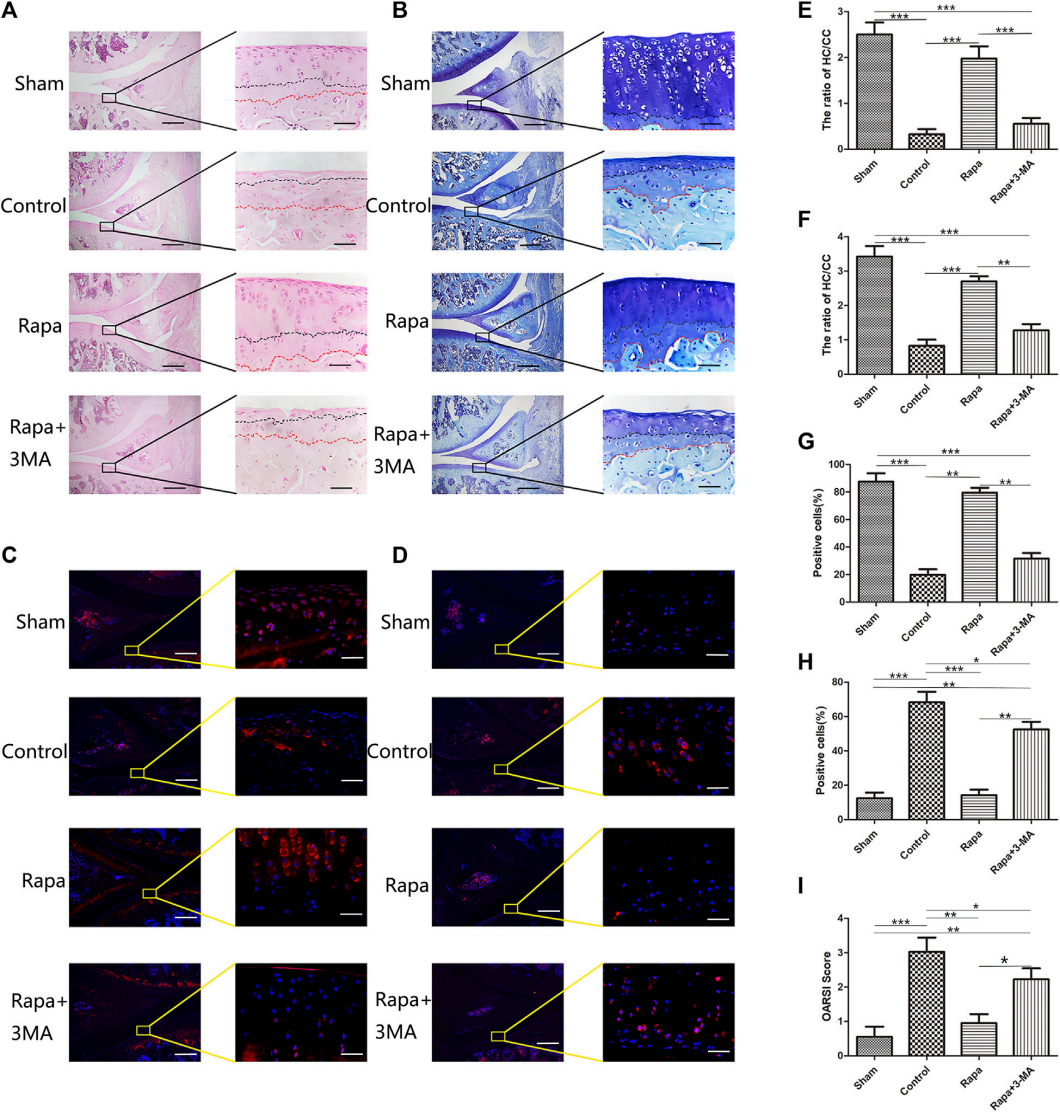
Histological and immunofluorescence staining of intra-articular injection of rapamycin **(A)**. The result of HE staining (Scale bar: the left panel was 200 um, the right panel was 25 um) **(B)**. The result of Toluidine blue staining (Scale bar: the left panel was 200 um, the right panel was 25 um) **(C)**. The immunofluorescence results of Col2 (Scale bar: the left panel was 200 um, the right panel was 25 um) **(D)**. The immunofluorescence results of MMP13 (Scale bar: the left panel was 200um, the right panel was 25 um) **(E)**. HC/CC ratio under HE staining **(F)**. HC/CC ratio under toluidine blue staining **(G)**. The statistics of Col2 -positive cells **(H)**. The statistics of MMP13-positive cells. **(I)**. OARSI score in each group.

From the results of HE staining (Figure 1A), we can find that the ratio of hyaline cartilage and calcified cartilage (HC/CC) on the tibial side is the worst in the Control group, the ratio is around 0.2, and the ratio of HC/CC in the Rapa+3-MA group was close to that of the Control group, and the ratio was around 0.4. In sharp contrast, we could find that the ratio of HC/CC was significantly increased in the Rapa group, and it reached around 2.0. The Sham group had the highest ratio, reaching around 2.5 (Figure 1E). The toluidine blue staining results showed the same trend as the HE staining results (Figures 1B,F). Then we performed immunofluorescence staining to observe the expression of Col2 and MMP13 on the tibial side. We could find that Col2 expression in the Control group and the Rapa + 3-MA group was very low, significantly lower than that in the Rapa group, while the expression in the Rapa group was slightly lower than that in the Sham group (Figures 1C,G). In contrast, we could find that the expression of MMP13 was higher in the Control group and the Rapa + 3-MA group, which was significantly higher than that in the Rapa group, while the expression in the Rapa group was similar to that in the Sham group (Figures 1D,H). Finally, combined with the OARSI score of osteoarthritis (Figure 1I; Supplementary Table S2), we can find that rapamycin can indeed reduce the expression of cartilage damage-related factor MMP13, protecting Col2 in cartilage tissue from catabolism factor-induced degradation, thereby delaying the progression of PTOA in ACLT rats.

### The mTORC1 signaling pathway was inhibited in the meniscus

**FIGURE 2:**
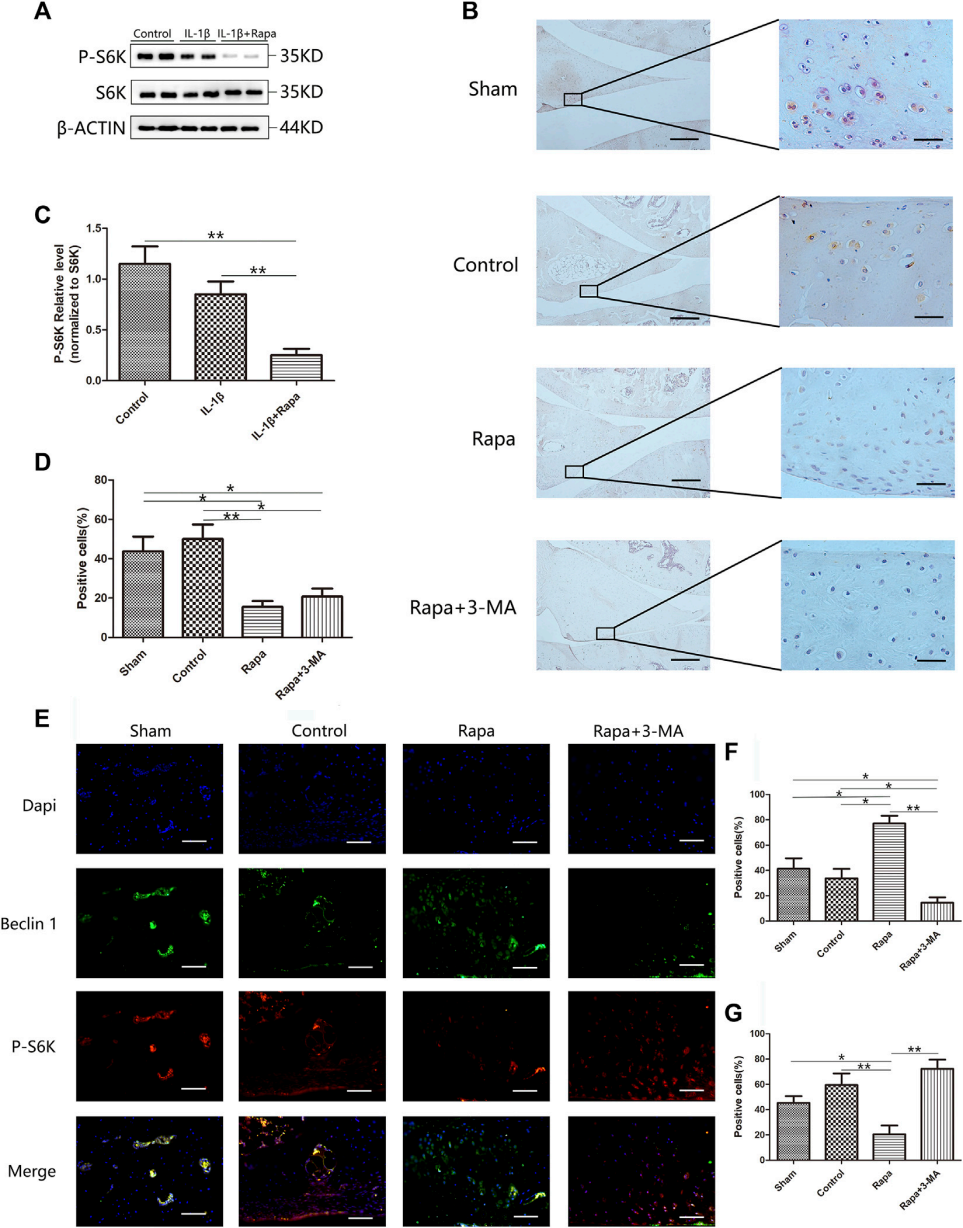
The expression of the mTORC1 signaling pathway in the meniscus **(A)**. The expression of S6K and P-S6K in Western blot **(B)**. The immunohistochemical staining of P-AKT (Scale bar: the left panel was 200 um, the right panel was 25 um) **(C)**. The gray value statistics of P-S6K in Western blot **(D)**. The statistics of P-AKT-positive cells **(E)**. The immunofluorescence co-staining results of P-S6K and Beclin 1 (Scale bar: 50 um) **(F)**. The statistics of Beclin 1-positive cells **(G)**. The statistics of P-S6K-positive cells.

To investigate whether rapamycin can affect the mTORC1 signaling pathway in the meniscus, we used western blot to verify the expression of S6K and P-S6K in each group. We found that rapamycin can significantly inhibit the expression of P-S6K in meniscal cells. Phosphorylation of ribosomal protein S6K is one of the key steps in the activation of the mTORC1 signaling pathway. Inhibiting the expression of P-S6K indicates that rapamycin can well inhibit mTORC1 activity in meniscal cells (Figures 2A,C). Further, we used immunohistochemical staining to verify the expression of P-AKT in the meniscus tissue. We could find that the expression of P-AKT was higher in both the Sham group and the Control group, which was significantly higher than in the Rapa group and the Rapa+3-MA group. As another key molecule in the mTORC1 signaling pathway, the decreased expression of P-AKT indicating that intra-articular injection of rapamycin can also effectively inhibit the mTORC1 signaling pathway in meniscus tissue (Figures 2B,D). Finally, we performed an immunofluorescence co-staining on the expression of P-S6K and Beclin 1 in the meniscus tissue, and we could find the expression of P-S6K in the Control group and the Rapa+3-MA group higher than the Sham group and the Rapa group, in which the expression was the lowest in the Rapa group. Interestingly, the expression of Beclin 1 was opposite to that of P-S6K, and the expression of Beclin 1 in the Control group and the Rapa+3-MA group was much lower than that in the Sham group and the Rapa group, where Expression was highest in the Rapa group (Figures 2E,F,G).

### Autophagy-related signaling pathway was activated in the meniscus

**FIGURE 3:**
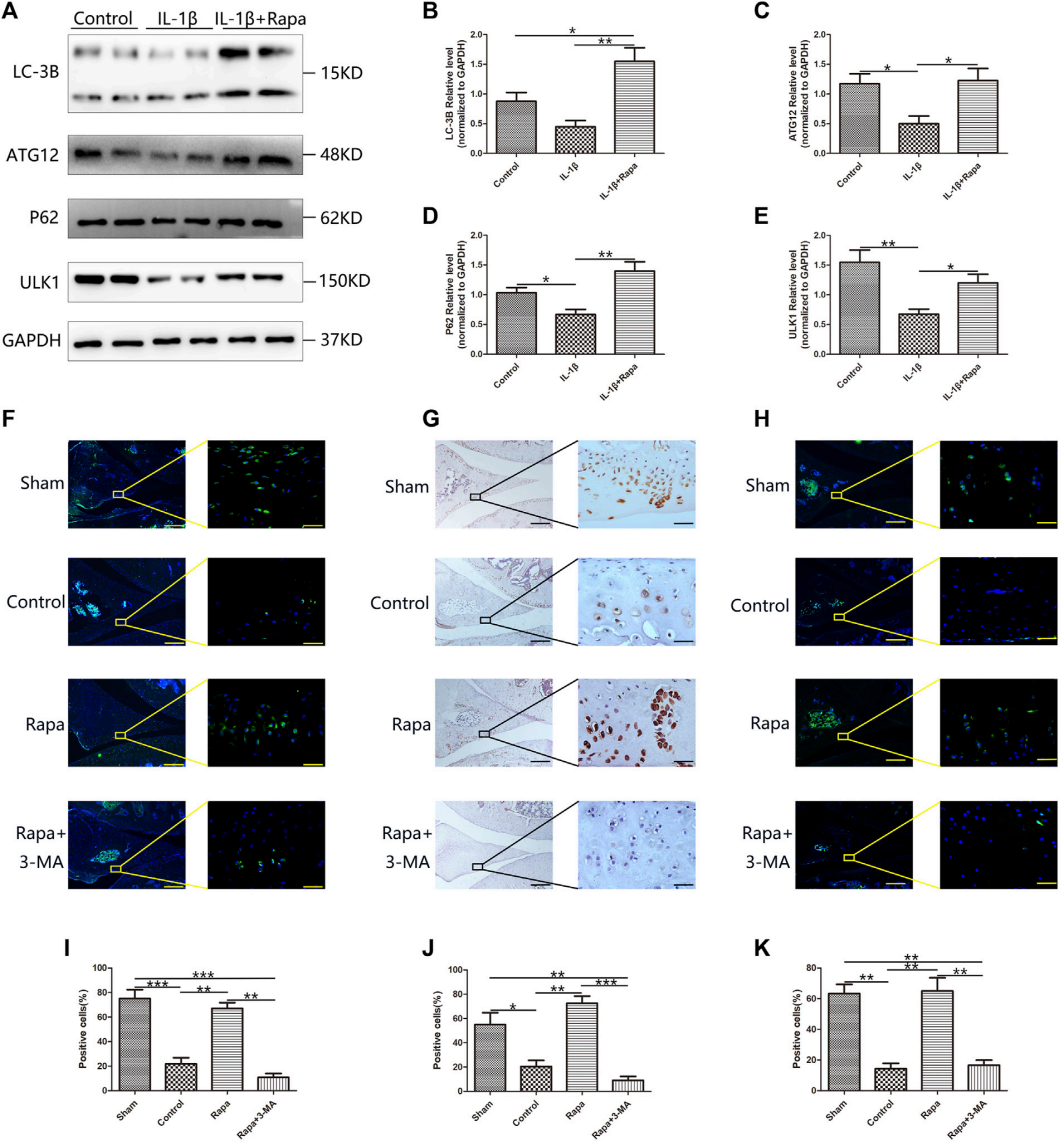
The expression of autophagy-related signaling pathways in the meniscus **(A)**. The expression of LC-3B, ATG12, P62 and ULK1 in Western Blot **(B)**. The gray value statistics of LC-3B **(C)**. The gray value statistics of ATG12 **(D)**. The gray value statistics of P62. **(E)**. The gray value statistics of ULK1. **(F)** The immunofluorescence staining results of ATG12 (Scale bar: the left panel was 200 um, the right panel was 25 um) **(G)**. The immunohistochemical staining results of LC-3B (Scale bar: the left panel was 200 um, the right panel was 25 um) **(H)**. The immunofluorescence staining results of P62 (Scale bar: the left panel was 200um, the right panel was 25 um) **(I)**. The statistics of ATG12-positive cells **(J)**. The statistics of LC-3B-positive cells **(K)**. The statistics of P62-positive cells.

Western blotting verified the expression of LC-3B, ATG12, P62 and ULK1 in each group of cells, we could find that rapamycin could significantly promote the expression of LC-3B, ATG12, P62, and ULK1. LC-3B, ATG12, P62 and ULK1 are key components of autophagy-related signaling pathways and promoting the expression of LC-3B, ATG12, P62, and ULK1 indicate that rapamycin can well promote autophagy-related signaling pathways in meniscal cells (Figures 3A–E). Then we used immunofluorescence staining and immunohistochemical staining to observe the expression of ATG12 (Figures 3F,I), LC-3B (Figures 3G,J), and P62 (Figures 3H,K) in the meniscus tissue respectively, and we could find that the expressions of ATG12, LC-3B, and P62 in the Sham group and the Rapa groups were significantly higher than those in the Control group and the Rapa + 3-MA group, and the expression in the Rapa + 3-MA group was the lowest. As important components of autophagy-related signaling pathways, the elevated expressions of ATG12, LC-3B and P62 indicated that intra-articular injection of rapamycin could well promote autophagy-related signaling pathways in meniscus tissue.

### Energy metabolism was enhanced in the meniscus and its apoptosis was inhibited

**FIGURE 4:**
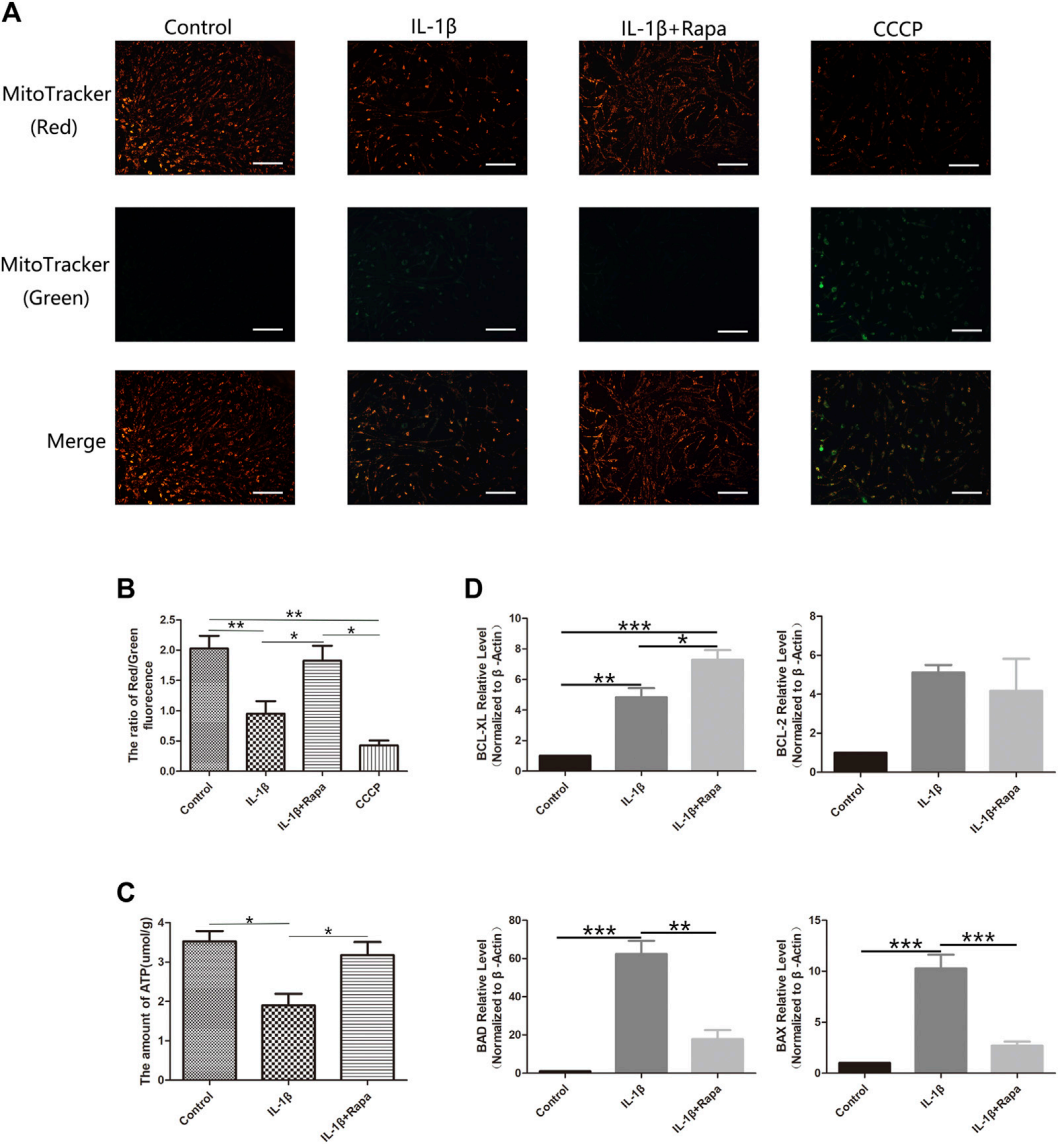
Energy metabolism and the expression of apoptosis-related factors in meniscal cells **(A)**. The result of Mitochondrial membrane potential staining (Scale bar: 50 um) **(B)**. The red/green fluorescence ratio of mitochondrial membrane potential staining results **(C)**. The statistics of ATP content detection **(D)**. The statistics of RT-qPCR detection results.

After rapamycin inhibits the mTORC1 signaling pathway and activates the autophagy-related signaling pathway in the meniscus, we further detected the changes in mitochondrial membrane potential in each group of cells with the mitochondrial membrane potential detection kit. Compared with the Control group, the IL-1β group can significantly reduce the mitochondrial membrane potential in meniscal cells, while rapamycin can rescue and increase the mitochondrial membrane potential in meniscal cells (Figures 4A,B). In addition, we also used an ATP content detection kit to detect the ATP content in the meniscus cells of the corresponding groups, we also found the same situation with the mitochondrial membrane potential: the ATP content can be significantly reduced in meniscal cells in IL-1β group compared with the Control group, which can be rescued by rapamycin to increase the content of ATP in meniscus cells (Figure 4C). Finally, we used RT-qPCR experiments to detect apoptosis-related factors such as BCL- XL, BCL-2, BAD, and BAX at the transcriptional level. We can find that the IL-1β group can simultaneously increase the expression of anti-apoptotic factors BCL-XL, BCL-2, pro-apoptotic factors BAD and BAX compared with the Control group. But the expression level of BCL-XL increased more obviously in the IL-1β+Rapa group, and it also decreased the expression of pro-apoptotic factors BAD and BAX (Figure 4D). Therefore, rapamycin inhibits the mTORC1 signaling pathway and activating the autophagy-related signaling pathway, it can increase the mitochondrial membrane potential and the ATP content in the meniscus cells affected by the inflammatory factor IL-1β, thereby reducing apoptosis of meniscus cells.

### The extracellular matrix damage of the meniscus was reduced

**FIGURE 5:**
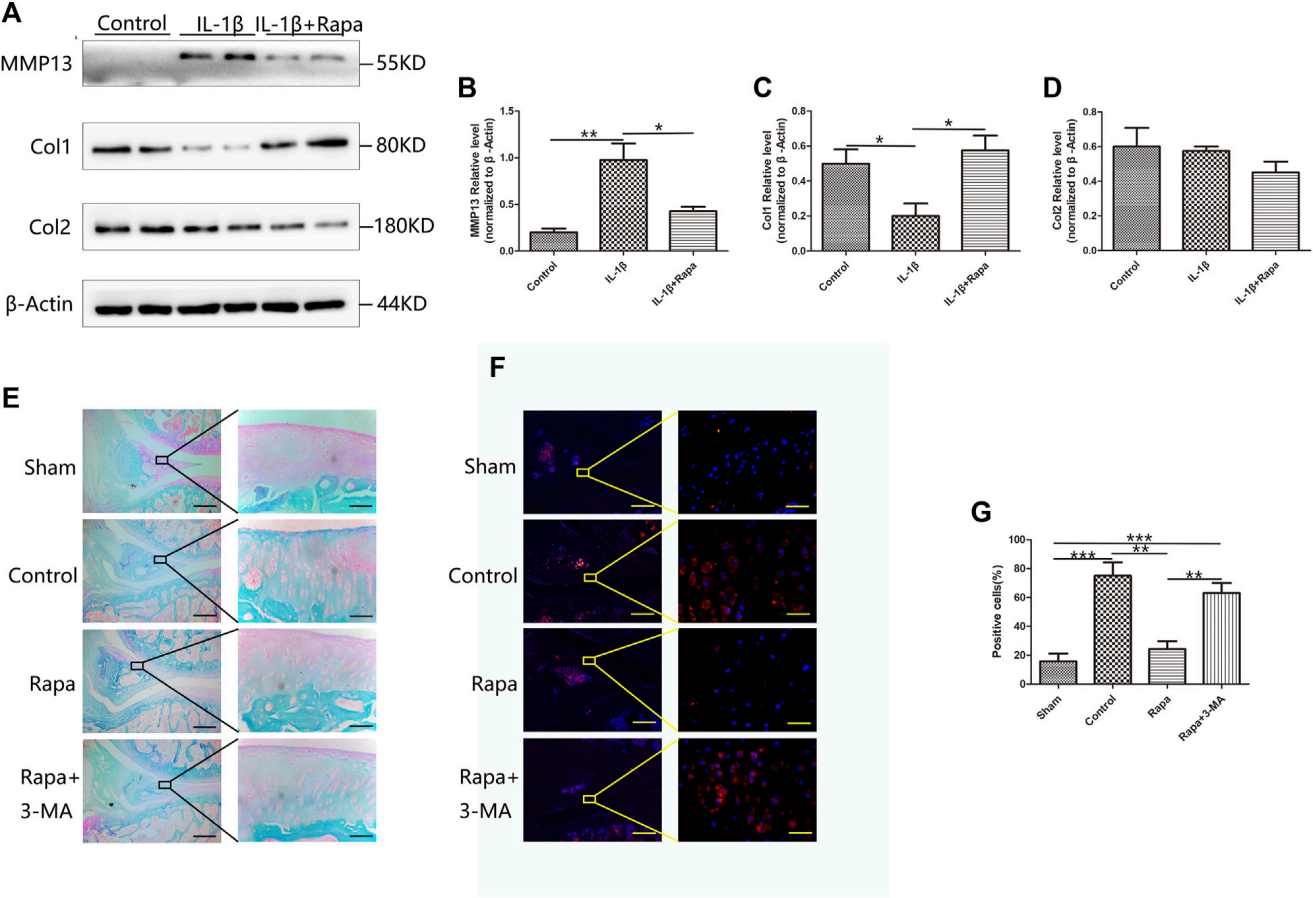
The protective effect of rapamycin on the meniscus extracellular matrix. **(A)** The expression of MMP13, Col1, and Col2 in Western Blot **(B)**. The gray value statistics of MMP13 **(C)**. The gray value statistics of Col1 **(D)**. The gray value statistics of Col2 **(E)**. The result of safranin O/fast green staining (Scale bar: the left panel was 200 um, the right panel was 25 um) **(F)**. The immunofluorescence staining results of MMP13 (Scale bar: the left panel was 200 um, the right panel was 25 um) **(G)**. The statistics of MMP13-positive cells.

At the cellular level, we detected the expression of MMP13, Col1 and Col2 in each experimental group by Western Blot. In contrast, IL-1β could significantly increase the expression of MMP13 in meniscal cells, resulting in a decrease in the expression of Col1 and Col2. Rapamycin can reduce the increased expression of MMP13 caused by IL-1β in meniscal cells, thereby protecting Col1 in meniscal cells from degradation, but it has little effect on the protection of Col2 in meniscal cells (Figures 5A–D). Then we used safranin O/fast green staining to observe the meniscus tissue. We found that the cartilage components in the meniscus of the Sham group were the most intact, followed by the Rapa group. However, the most of the cartilage components in the Control group and Rapa+3 MA group were destroyed (Figure 5E). We also used immunofluorescence staining to observe the MMP13 in the meniscus tissue. We found that the expression of MMP13 in the Rapa group was significantly lower than that of the Control group and Rapa+3 MA *in vivo*, and was similar to the Sham group (Figures 5F,G). Therefore, unlike articular cartilage, we believe that rapamycin protects meniscal cells by protecting Col1 from degradation by MMP13.

### Meniscus damage prior to articular cartilage in anterior cruciate ligament transection rats

**FIGURE 6:**
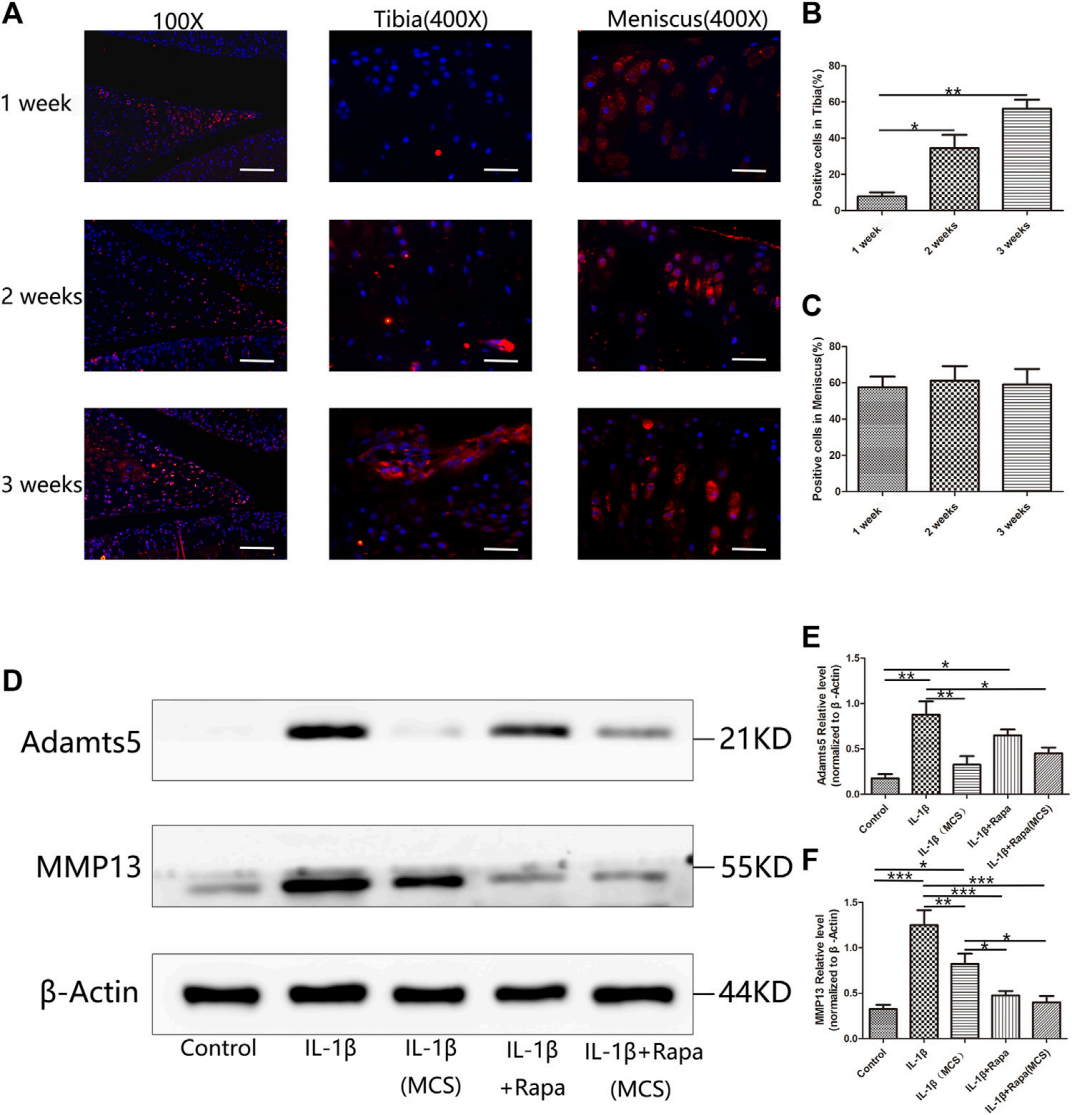
Influence of the meniscus on articular cartilage during the development of PTOA **(A)**. The immunofluorescence staining results of MMP13 in 1 week, 2 and 3 weeks after ACLT surgery (Scale bar: the left panel was 100 um, the middle and the right panel was 25 um). **(B)** The statistics of MMP13-positive cells in tibia **(C)**. The statistics of MMP13-positive cells in meniscus **(D)**. The expression of Adamts5 and MMP13 in Western Blot **(E)**. The gray value statistics of Adamts5 **(F)** The gray value statistics of MMP13.

During clinical arthroscopic surgery, we often found that meniscal tissue always has morphological changes prior to articular cartilage in patients with only ACLT (Supplementary Figure S2). To find out whether the damage to the meniscus prior to the articular cartilage in the development of PTOA, and whether the meniscus not only affects the articular cartilage from a biomechanical perspective but also interacts with the articular cartilage from a biochemical perspective, we designed an animal experiment, samples were collected at 1 week, 2 and 3 weeks after ACLT modeling in SD rats for immunofluorescence staining and the expression of MMP13 in the meniscus and articular cartilage was observed. We can find that MMP13 has been abundantly expressed on the meniscus tissue at 1 week, while the expression in the tibial articular cartilage is little at this time. As time progresses, MMP13 is increased expression in the tibial articular cartilage at 2 and 3 weeks, while the expression of MMP13 in the meniscus tissue has been in a high expression state (Figures 6A–C). To study the effect of meniscus injury on articular cartilage, we then designed a cell co-culture experiment, using IL-1β to directly stimulate chondrocytes and IL-1β to stimulate meniscal cells and then use meniscus culture supernatant (MCS) culture chondrocytes. The expression of MMP13 and Adamts5 in chondrocytes was observed. We found that chondrocytes in the MCS could significantly reduce the expression of MMP13 and Adamts5 induced by IL-1β and this effect can be enhanced by rapamycin (Figures 6D–F). Combined with the above experimental results, we found that in the development of PTOA after ACLT, the damage of meniscus tissue will take prior to articular cartilage. The meniscus tissue is not only a biomechanical mediator of articular cartilage damage influenced by IL-1β, and biochemically, the meniscus tissue will protect the articular cartilage to a certain extent by endocrine protective factors.

## Conclusion

OA is the most prevalent chronic deforming joint disease. Progressive painful osteoarthritis can lead to severe joint dysfunction that increases the risk of all-cause mortality. Age, gender, mechanical stress and genetic factors are considered to be risk factors for the development of OA. Further understanding of the relevant molecular mechanisms is crucial for exploring new therapeutic strategies in the OA process. The pathogenesis of OA is multifactorial and mutually regulated by several signaling pathways, which in turn promote the development of OA (Rezus et al., 2021). Recent studies have reported that increased inflammatory factors such as IL-1β, TNF-α, and IL-6 in OA cartilage contribute to the pathogenesis of OA, which leads to the occurrence of OA disease. Accumulating evidence suggests that IL-1β, one of the important inflammatory cytokines, accelerates chondrocyte catabolism by increasing ECM degradation by upregulating MMPs. Overproduction of cytokines and growth factors has been shown to play an important role in the pathophysiology of OA. In addition, inflammatory factors such as IL-1β and TNF-α have been shown to contribute to chondrocyte mitochondrial dysfunction and associated apoptosis. The IL-1 family of cytokines is a key regulator of infectious or sterile inflammatory responses. Inflammatory IL-1β, the most important cytokine, is directly involved in the production of multiple inflammatory mediators (such as TNF-α, IL-6, and MMPs), disrupting the balance of ECM metabolism. The expression of IL-1β in normal human serum is low, whereas local production of IL-1β in OA cartilage induces further downstream mediators, which then play a regulatory role in multiple pathological processes of OA. There is some compelling evidence that the inflammation-related changes in OA cartilage are due to the activation of NF-κB signaling in chondrocytes (Lepetsos et al., 2019; Zhao et al., 2019; Li et al., 2020; Liao et al., 2020). Therefore, inhibiting these inflammatory mediators or disrupting inflammatory pathways, and reducing inflammation-related apoptosis may be effective in the treatment of OA.

The meniscus plays an important role in knee load transmission, shock absorption, and mechanical stability, biomechanical functions that protect articular cartilage from degeneration and damage (Cavanaugh, 2014; Bryceland et al., 2017). Although articular cartilage degeneration is the main pathogenesis of OA, meniscal degeneration often accompanies the occurrence and development of OA, and there is more literal support that the meniscus is already damaged in the early stage of OA (Favero et al., 2019). Meniscal injury has become a significant risk factor for OA, and knee meniscus injury and degeneration are closely related to the development of OA. More than 75% of OA patients also suffer from meniscal lesions. 50% of patients with meniscal damage also develop PTOA within 10–20 years (Xu et al., 2021). Although the exact pathogenic process of meniscal degeneration is unknown. Injury or destruction of the meniscus disrupts biomechanical and biochemical properties and is associated with cell death, abnormal cell activation and differentiation (Englund et al., 2009). The meniscal injury also disrupts tissue homeostasis, leading to progressive articular cartilage extracellular matrix (ECM) degeneration, which ultimately manifests as OA throughout the joint (Park et al., 2021).

Autophagy is an important cellular homeostasis mechanism that supports normal cellular function and survival under stress-induced conditions. This catabolic process regulates energy and nutrition by removing damaged or dysfunctional proteins and organelles (Glick et al., 2010). Excessive accumulation of dysfunctional proteins and damaged organelles can be found by the defects of autophagy, ultimately leading to cell death. Aging increases susceptibility to defects in autophagy, which have been implicated in many pathogenic processes, including cancer and neurodegeneration (Levine and Kroemer, 2019; Klionsky et al., 2021). In OA, autophagy defects in articular cartilage are associated with cartilage degeneration. In articular cartilage tissue, resident chondrocytes cannot be replenished through the vasculature, so autophagy is essential for maintaining cellular and tissue homeostasis (Musumeci et al., 2015; Duan et al., 2020). In humans and mice, the expression of autophagy proteins ATG-5 and LC-3B in articular cartilage both decreased with age. These changes were accompanied by a decrease in cell numbers and an increase in apoptotic cell death as well as degradation of the ECM and development of OA (Xue et al., 2017; Wang et al., 2020). When DMM-modeled mice were treated with rapamycin, a pharmacological activator of autophagy, the cellular identity of chondrocytes was preserved, preventing more severe damage and degeneration of articular cartilage. Taken together, these findings suggest that articular chondrocyte autophagy plays an important role in joint aging and the development of OA (Zhang et al., 2015; Meckes et al., 2017). Autophagy may also play a role in meniscal injury and degeneration, and PTOA following meniscal injury. However, little is known about the role of autophagy in meniscus health, injury, and degeneration, and the pathogenesis of PTOA following meniscal injury is rarely reported.

In the process of cellular energy metabolism, ATP is not only an important energy storage material but also a direct energy supply material. When the tissue cells of the body carry out various functional activities, the direct source of energy is the stored energy in ATP (Hargreaves and Spriet, 2018). In addition to ATP, there is another energy storage compound containing high-energy phosphate bonds in the body, namely creatine phosphate (CP). When the energy generated by the decomposition of substances in the body increases and the concentration of ATP formed increases, ATP will transfer the high-energy phosphate bond to creatine to generate CP to store the energy. On the contrary, when the energy consumption of tissue cells increases and the ATP concentration decreases, The stored energy is then transferred to adenosine diphosphate (ADP) to generate new ATP. Therefore, CP is often regarded as a reservoir of ATP (Loike et al., 1979; Strumia et al., 2012). From the perspective of the entire process of energy metabolism, the synthesis and decomposition of ATP is the key link in the conversion and utilization of cellular energy in the body (Manna et al., 2020). When meniscal cells are stimulated by inflammatory factors such as IL-1β, TNF-α, etc., the energy metabolism of meniscus cells and the relationship between the changes in energy metabolism and PTOA have not been reported.

Through our research, we found that rapamycin can inhibit the phosphorylation of S6K and AKT in the meniscus, thereby inhibiting the activation of the mTORC1 signaling pathway in the meniscus. The increased expression of autophagy-related molecules such as LC-3B, ATG12, P62, and ULK1 further reduces the expression of MMP13 in the meniscus, and protects the main component of the meniscus tissue. Subsequently, rapamycin rescued the decreased energy production of meniscal cells by the inflammatory factor IL-1β, inhibiting meniscal cell apoptosis. We also found that the meniscus expresses a greater amount of MMP13 before the articular cartilage in the development of PTOA, which means that the damage of the meniscus prior to the articular cartilage in the development of PTOA. After the injury, protective cytokines are produced to a certain extent to protect articular cartilage from damage, thereby delaying the progression of PTOA in ACLT rats.

It is worth mentioning that this study still has its shortcomings. The first is that the cytokines released by the meniscus tissue during the transduction process of abnormal mechanical signals need to be further studied. The second is the impaired autophagy mediated by the activated mTORC1 signaling pathway during abnormal transduction can accelerate the cartilage damage caused by the abnormal meniscus, which is only one of the signaling pathways in the mechanical signal transduction process, and other related signaling pathways need to be further studied. Finally, the new treatment strategies needs to be innovated after the meniscus is damaged, to better prevent the deformation of the meniscus and the occurrence and development of PTOA.

In conclusion, this study illustrates the role between the meniscus and articular cartilage in mediating the effects of PTOA. On the one hand, the results of experiments *in vitro* show that rapamycin can activate autophagy-related signaling pathways by inhibiting the mTORC1 signaling pathway in meniscal cells, thereby protecting the extracellular matrix components of the meniscus, promoting energy production and inhibiting apoptosis of meniscal cells. On the other hand, experimental results *in vivo* show that rapamycin can activate autophagy-related signaling pathways by inhibiting the mTORC1 signaling pathway in the meniscus tissue to protect the extracellular matrix components of the meniscus, thereby delaying the occurrence and development PTOA caused by secondary articular cartilage damage. Therefore, the regulation of autophagy mediated by the mTORC1 signaling pathway in meniscus tissue can delay the secondary cartilage damage caused by meniscal abnormalities. Enhancing autophagy and energy metabolism in the meniscus will be expected as a new therapeutic approach to prevent meniscal degeneration and promoting meniscus repair, thereby delaying the occurrence of PTOA.

## Data Availability

The raw data supporting the conclusions of this article will be made available by the authors, without undue reservation.

## References

[B1] Ben-Sahra I. Manning B. D. (2017). Mtorc1 signaling and the metabolic control of cell growth. Curr. Opin. Cell. Biol. 45, 72–82. PubMed Abstract | 10.1016/j.ceb.2017.02.012 | Google Scholar 28411448 PMC5545101

[B2] Bryceland J. K. Powell A. J. Nunn T. (2017). Knee menisci. Cartilage 8, 99–104. 10.1177/1947603516654945 PubMed Abstract | 10.1177/1947603516654945 | Google Scholar 28345407 PMC5358830

[B3] Butterfield N. C. Curry K. F. Steinberg J. Dewhurst H. Komla-Ebri D. Mannan N. S. (2021). Publisher Correction: Accelerating functional gene discovery in osteoarthritis. Nat. Commun. 12, 3302. 10.1038/s41467-021-23768-8 PubMed Abstract | 10.1038/s41467-021-23768-8 | Google Scholar 34050183 PMC8163861

[B4] Cavanaugh J. T. (2014). Rehabilitation of meniscal injury and surgery. J. Knee Surg. 27, 459–478. 10.1055/s-0034-1394299 PubMed Abstract | 10.1055/s-0034-1394299 | Google Scholar 25390473

[B5] Duan R. Xie H. Liu Z. Z. (2020). The role of autophagy in osteoarthritis. Front. Cell. Dev. Biol. 8, 608388. 10.3389/fcell.2020.608388 PubMed Abstract | 10.3389/fcell.2020.608388 | Google Scholar 33324654 PMC7723985

[B6] Englund M. Guermazi A. Lohmander S. L. (2009). The role of the meniscus in knee osteoarthritis: A cause or consequence? Radiol. Clin. North Am. 47, 703–712. 10.1016/j.rcl.2009.03.003 PubMed Abstract | 10.1016/j.rcl.2009.03.003 | Google Scholar 19631077

[B7] Favero M. Belluzzi E. Trisolino G. Goldring M. B. Goldring S. R. Cigolotti A. (2019). Inflammatory molecules produced by meniscus and synovium in early and end-stage osteoarthritis: A coculture study. J. Cell. Physiol. 234, 11176–11187. 10.1002/jcp.27766 PubMed Abstract | 10.1002/jcp.27766 | Google Scholar 30456760

[B8] Glick D. Barth S. Macleod K. F. (2010). Autophagy: Cellular and molecular mechanisms. J. Pathol. 221, 3–12. 10.1002/path.2697 PubMed Abstract | 10.1002/path.2697 | Google Scholar 20225336 PMC2990190

[B9] Griffin T. M. Scanzello C. R. (2019). Innate inflammation and synovial macrophages in osteoarthritis pathophysiology. Clin. Exp. Rheumatol. 37 (120), 57–63. PubMed Abstract | Google Scholar PMC6842324 31621560

[B10] Hargreaves M. Spriet L. L. (2018). Exercise metabolism: Fuels for the fire. Cold Spring Harb. Perspect. Med. 8, a029744. 10.1101/cshperspect.a029744 PubMed Abstract | 10.1101/cshperspect.a029744 | Google Scholar 28533314 PMC6071548

[B11] Hayami T. Zhuo Y. Wesolowski G. A. Pickarski M. Duong L. T. (2012). Inhibition of cathepsin K reduces cartilage degeneration in the anterior cruciate ligament transection rabbit and murine models of osteoarthritis. Bone 50, 1250–1259. 10.1016/j.bone.2012.03.025 PubMed Abstract | 10.1016/j.bone.2012.03.025 | Google Scholar 22484689

[B12] Housmans B. A. C. Neefjes M. Surtel D. A. M. Vitik M. Cremers A. Van Rhijn L. W. (2022). Synovial fluid from end-stage osteoarthritis induces proliferation and fibrosis of articular chondrocytes via mapk and rhogtpase signaling. Rheumatology: Osteoarthritis Cartilage. Google Scholar 10.1016/j.joca.2021.12.015 35176481

[B13] Hu Y. Chen X. Wang S. Jing Y. Su J. (2021). Subchondral bone microenvironment in osteoarthritis and pain. Bone Res. 9, 20. 10.1038/s41413-021-00147-z PubMed Abstract | 10.1038/s41413-021-00147-z | Google Scholar 33731688 PMC7969608

[B14] Judge A. Dodd M. S. (2020). Metabolism. Essays Biochem. 64, 607–647. 10.1042/EBC20190041 PubMed Abstract | 10.1042/EBC20190041 | Google Scholar 32830223 PMC7545035

[B15] Klionsky D. J. Petroni G. Amaravadi R. K. Baehrecke E. H. Ballabio A. Boya P. (2021). Autophagy in major human diseases. Embo J. 40, E108863. 10.15252/embj.2021108863 PubMed Abstract | 10.15252/embj.2021108863 | Google Scholar 34459017 PMC8488577

[B16] Koushesh S. Shahtaheri S. M. Mcwilliams D. F. Walsh D. A. Sheppard M. N. Westaby J. (2022). The osteoarthritis bone score (oabs): A new histological scoring system for the characterisation of bone marrow lesions in osteoarthritis. Rheumatology: Osteoarthritis Cartilage. Google Scholar 10.1016/j.joca.2022.01.008 PMC9395274 35124198

[B17] Lee P. Chandel N. S. Simon M. C. (2020). Cellular adaptation to hypoxia through hypoxia inducible factors and beyond. Nat. Rev. Mol. Cell. Biol. 21, 268–283. 10.1038/s41580-020-0227-y PubMed Abstract | 10.1038/s41580-020-0227-y | Google Scholar 32144406 PMC7222024

[B18] Lepetsos P. Papavassiliou K. A. Papavassiliou A. G. (2019). Redox and NF-κB signaling in osteoarthritis. Free Radic. Biol. Med. 132, 90–100. 10.1016/j.freeradbiomed.2018.09.025 PubMed Abstract | 10.1016/j.freeradbiomed.2018.09.025 | Google Scholar 30236789

[B19] Levine B. Kroemer G. (2019). Biological functions of autophagy genes: A disease perspective. Cell. 176, 11–42. 10.1016/j.cell.2018.09.048 PubMed Abstract | 10.1016/j.cell.2018.09.048 | Google Scholar 30633901 PMC6347410

[B20] Li Z. Cheng J. Liu J. (2020). Baicalin protects human OA chondrocytes against IL-1β-induced apoptosis and ECM degradation by activating autophagy via MiR-766-3p/AIFM1 Axis. Drug Des. devel. Ther. 14, 2645–2655. 10.2147/DDDT.S255823 PubMed Abstract | 10.2147/DDDT.S255823 | Google Scholar PMC7353997 32753846

[B21] Liao C. R. Wang S. N. Zhu S. Y. Wang Y. Q. Li Z. Z. Liu Z. Y. (2020). Advanced oxidation protein products increase TNF-α and IL-1β expression in chondrocytes via NADPH oxidase 4 and accelerate cartilage degeneration in osteoarthritis progression. Redox Biol. 28, 101306. 10.1016/j.redox.2019.101306 PubMed Abstract | 10.1016/j.redox.2019.101306 | Google Scholar 31539804 PMC6812020

[B22] Liu G. Y. Sabatini D. M. (2020). Mtor at the nexus of nutrition, growth, ageing and disease. Nat. Rev. Mol. Cell. Biol. 21, 183–203. 10.1038/s41580-019-0199-y PubMed Abstract | 10.1038/s41580-019-0199-y | Google Scholar 31937935 PMC7102936

[B23] Liu H. Zhu H. Cheng L. Zhao Y. Chen X. Li J. (2021). Tcp/plga composite scaffold loaded rapamycin *in situ* enhances lumbar fusion by regulating osteoblast and osteoclast activity. J. Tissue Eng. Regen. Med. 15, 475–486. 10.1002/term.3186 PubMed Abstract | 10.1002/term.3186 | Google Scholar 33686790

[B24] Loike J. D. Kozler V. F. Silverstein S. C. (1979). Increased atp and creatine phosphate turnover in phagocytosing mouse peritoneal macrophages. J. Biol. Chem. 254, 9558–9564. 10.1016/s0021-9258(19)83551-2 PubMed Abstract | 10.1016/s0021-9258(19)83551-2 | Google Scholar 489550

[B25] Lorenz J. Grassel S. (2014). Experimental osteoarthritis models in mice. Methods Mol. Biol. 1194, 401–419. 10.1007/978-1-4939-1215-5_23 PubMed Abstract | 10.1007/978-1-4939-1215-5_23 | Google Scholar 25064117

[B26] Lunt S. Y. Vander Heiden M. G. (2011). Aerobic glycolysis: Meeting the metabolic requirements of cell proliferation. Annu. Rev. Cell. Dev. Biol. 27, 441–464. 10.1146/annurev-cellbio-092910-154237 PubMed Abstract | 10.1146/annurev-cellbio-092910-154237 | Google Scholar 21985671

[B27] Macfarlane E. G. Haupt J. Dietz H. C. Shore E. M. (2017). Tgf-beta family signaling in connective tissue and skeletal diseases. Cold Spring Harb. Perspect. Biol. 9, a022269. 10.1101/cshperspect.a022269 PubMed Abstract | 10.1101/cshperspect.a022269 | Google Scholar 28246187 PMC5666637

[B28] Makris E. A. Hadidi P. Athanasiou K. A. (2011). The knee meniscus: Structure-function, pathophysiology, current repair techniques, and prospects for regeneration. Biomaterials 32, 7411–7431. 10.1016/j.biomaterials.2011.06.037 PubMed Abstract | 10.1016/j.biomaterials.2011.06.037 | Google Scholar 21764438 PMC3161498

[B29] Malemud C. J. (2019). Inhibition of mmps and adam/adamts. Biochem. Pharmacol. 165, 33–40. 10.1016/j.bcp.2019.02.033 PubMed Abstract | 10.1016/j.bcp.2019.02.033 | Google Scholar 30826330 PMC6557692

[B30] Mandl L. A. (2019). Osteoarthritis year in review 2018: Clinical. Osteoarthr. Cartil. 27, 359–364. 10.1016/j.joca.2018.11.001 10.1016/j.joca.2018.11.001 | Google Scholar 30453055

[B31] Manna R. N. Dutta M. Jana B. (2020). Mechanistic study of the atp hydrolysis reaction in dynein motor protein. Phys. Chem. Chem. Phys. 22, 1534–1542. 10.1039/c9cp02194a PubMed Abstract | 10.1039/c9cp02194a | Google Scholar 31872818

[B32] Martel-Pelletier J. Barr A. J. Cicuttini F. M. Conaghan P. G. Cooper C. Goldring M. B. (2016). Osteoarthritis. Nat. Rev. Dis. Prim. 2, 16072. 10.1038/nrdp.2016.72 PubMed Abstract | 10.1038/nrdp.2016.72 | Google Scholar 27734845

[B33] Meckes J. K. Carames B. Olmer M. Kiosses W. B. Grogan S. P. Lotz M. K. (2017). Compromised autophagy precedes meniscus degeneration and cartilage damage in mice. Osteoarthr. Cartil. 25, 1880–1889. 10.1016/j.joca.2017.07.023 PubMed Abstract | 10.1016/j.joca.2017.07.023 | Google Scholar PMC5650923 28801209

[B34] Musumeci G. Castrogiovanni P. Trovato F. M. Weinberg A. M. Al-Wasiyah M. K. Alqahtani M. H. (2015). Biomarkers of chondrocyte apoptosis and autophagy in osteoarthritis. Int. J. Mol. Sci. 16, 20560–20575. 10.3390/ijms160920560 PubMed Abstract | 10.3390/ijms160920560 | Google Scholar 26334269 PMC4613218

[B35] Osterberg A. Thiem D. Herlyn P. Mittlmeier T. Frerich B. Muller-Hilke B. (2017). Subchondral bone sclerosis and cancellous bone loss following oa induction depend on the underlying bone phenotype. Jt. Bone Spine 84, 71–77. 10.1016/j.jbspin.2015.11.012 PubMed Abstract | 10.1016/j.jbspin.2015.11.012 | Google Scholar 27236261

[B36] Park J. Lee H. S. Go E. B. Lee J. Y. Kim J. Y. Lee S. Y. (2021). Proteomic analysis of the meniscus cartilage in osteoarthritis. Int. J. Mol. Sci. 22, 8181. 10.3390/ijms22158181 PubMed Abstract | 10.3390/ijms22158181 | Google Scholar 34360947 PMC8348647

[B37] Rabanal-Ruiz Y. Otten E. G. Korolchuk V. I. (2017). Mtorc1 as the main gateway to autophagy. Essays Biochem. 61, 565–584. 10.1042/EBC20170027 PubMed Abstract | 10.1042/EBC20170027 | Google Scholar 29233869 PMC5869864

[B38] Rezus E. Burlui A. Cardoneanu A. Macovei L. A. Tamba B. I. Rezus C. (2021). From pathogenesis to therapy in knee osteoarthritis: Bench-To-Bedside. Int. J. Mol. Sci. 22, 2697. 10.3390/ijms22052697 PubMed Abstract | 10.3390/ijms22052697 | Google Scholar 33800057 PMC7962130

[B39] Robinson W. H. Lepus C. M. Wang Q. Raghu H. Mao R. Lindstrom T. M. (2016). Low-grade inflammation as A key mediator of the pathogenesis of osteoarthritis. Nat. Rev. Rheumatol. 12, 580–592. 10.1038/nrrheum.2016.136 PubMed Abstract | 10.1038/nrrheum.2016.136 | Google Scholar 27539668 PMC5500215

[B40] Rodeo S. A. Monibi F. Dehghani B. Maher S. (2020). Biological and mechanical predictors of meniscus function: Basic science to clinical translation. J. Orthop. Res. 38, 937–945. 10.1002/jor.24552 PubMed Abstract | 10.1002/jor.24552 | Google Scholar 31799733

[B41] Strumia E. Pelliccia F. D'ambrosio G. (2012). Creatine phosphate: Pharmacological and clinical perspectives. Adv. Ther. 29, 99–123. 10.1007/s12325-011-0091-4 PubMed Abstract | 10.1007/s12325-011-0091-4 | Google Scholar 22297802

[B42] Suo J. Zou S. Wang J. Han Y. Zhang L. Lv C. (2022). The RNA-binding protein Musashi2 governs osteoblast-adipocyte lineage commitment by suppressing PPARγ signaling. Bone Res. 10, 31. 10.1038/s41413-022-00202-3 PubMed Abstract | 10.1038/s41413-022-00202-3 | Google Scholar 35301280 PMC8930990

[B43] Wang F. S. Kuo C. W. Ko J. Y. Chen Y. S. Wang S. Y. Ke H. J. (2020). Irisin mitigates oxidative stress, chondrocyte dysfunction and osteoarthritis development through regulating mitochondrial integrity and autophagy. Basel: Antioxidants. Google Scholar 10.3390/antiox9090810 PMC7555738 32882839

[B44] Wang T. He C. (2018). Pro-inflammatory cytokines: The link between obesity and osteoarthritis. Cytokine Growth Factor Rev. 44, 38–50. 10.1016/j.cytogfr.2018.10.002 PubMed Abstract | 10.1016/j.cytogfr.2018.10.002 | Google Scholar 30340925

[B45] Wen C. Xu L. Xu X. Wang D. Liang Y. Duan L. (2021). Insulin-like growth factor-1 in articular cartilage repair for osteoarthritis treatment. Arthritis Res. Ther. 23, 277. 10.1186/s13075-021-02662-0 PubMed Abstract | 10.1186/s13075-021-02662-0 | Google Scholar 34717735 PMC8556920

[B46] Wullschleger S. Loewith R. Hall M. N. (2006). Tor signaling in growth and metabolism. Cell. 124, 471–484. 10.1016/j.cell.2006.01.016 PubMed Abstract | 10.1016/j.cell.2006.01.016 | Google Scholar 16469695

[B47] Xu D. Van Der Voet J. Hansson N. M. Klein S. Oei E. H. G. Wagner F. (2021). Association between meniscal volume And development of knee osteoarthritis. Rheumatol. Oxf. 60, 1392–1399. 10.1093/rheumatology/keaa522 10.1093/rheumatology/keaa522 | Google Scholar PMC7937026 32974683

[B48] Xue J. F. Shi Z. M. Zou J. Li X. L. (2017). Inhibition of pi3k/akt/mtor signaling pathway promotes autophagy of articular chondrocytes and attenuates inflammatory response in rats with osteoarthritis. Biomed. Pharmacother. 89, 1252–1261. 10.1016/j.biopha.2017.01.130 PubMed Abstract | 10.1016/j.biopha.2017.01.130 | Google Scholar 28320092

[B49] Yan W. Xu X. Xu Q. Sun Z. Lv Z. Wu R. (2021). Chondral defects cause kissing lesions in A porcine model. Cartilage 13, 692s–702s. 10.1177/1947603520951636 PubMed Abstract | 10.1177/1947603520951636 | Google Scholar 32830514 PMC8804867

[B50] Zhang Y. Vasheghani F. Li Y. H. Blati M. Simeone K. Fahmi H. (2015). Cartilage-specific deletion of mtor upregulates autophagy and protects mice from osteoarthritis. Ann. Rheum. Dis. 74, 1432–1440. 10.1136/annrheumdis-2013-204599 PubMed Abstract | 10.1136/annrheumdis-2013-204599 | Google Scholar 24651621

[B51] Zhao Y. Li Y. Qu R. Chen X. Wang W. Qiu C. (2019). Cortistatin binds to tnf-alpha receptors and protects against osteoarthritis. Ebiomedicine 41, 556–570. 10.1016/j.ebiom.2019.02.035 PubMed Abstract | 10.1016/j.ebiom.2019.02.035 | Google Scholar 30826358 PMC6443028

