# Supplementary material for: Enhancing autophagy and energy metabolism in the meniscus can delay the occurrence of PTOA in ACLT rat
Source: Front Cell Dev Biol. 2022 Sep 2;10:971736. doi: 10.3389/fcell.2022.971736 (PMC9479128; doi:10.3389/fcell.2022.971736)
Supplement: Supplementary file 1 [file DataSheet1.PDF]

Supplemental information

Enhancing autophagy and energy metabolism in the meniscus can delay the occurrence of PTOA in ACLT rat

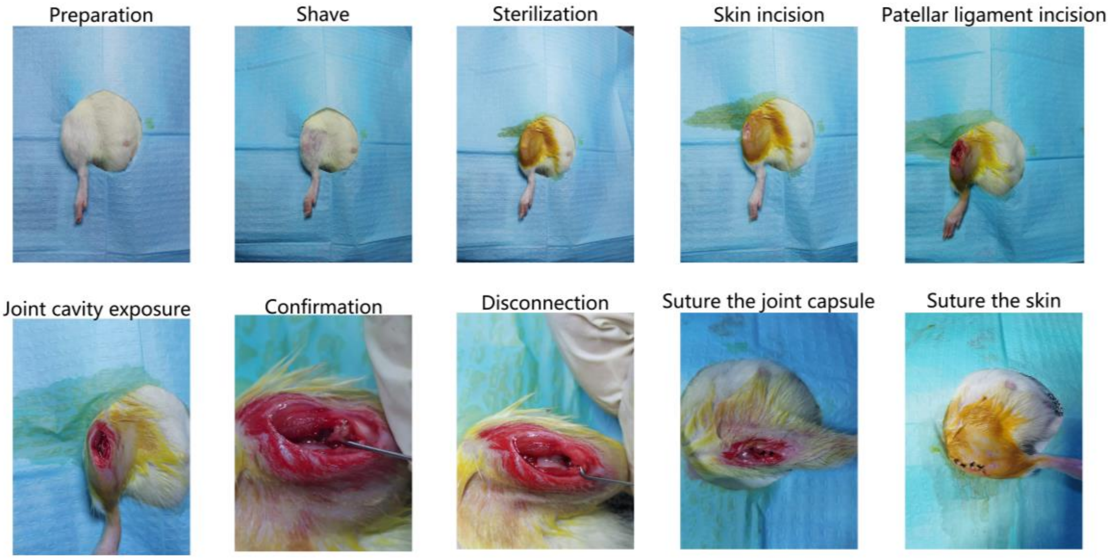

Figure S1. The detailed surgical procedure of anterior cruciate ligament transection (ACLT).

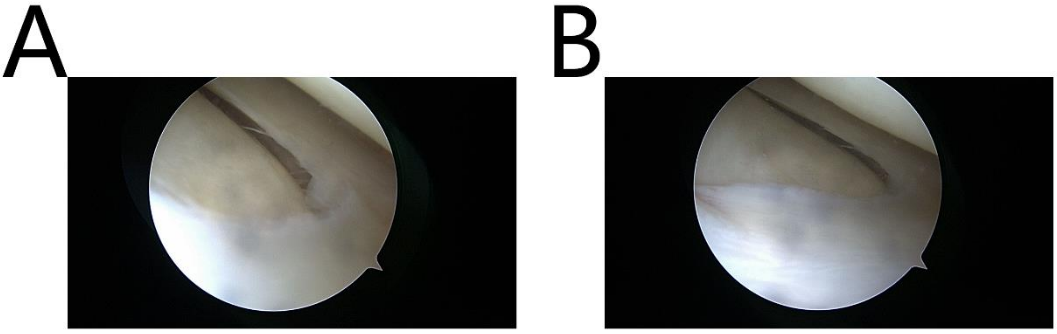

Figure S2. Image of clinical arthroscopy surgery. A. Image of patient 1. B. Image of patient 2.

Table S1. The specific primer sequences of RT-qPCR

Gene	Sequence
β-actin: (F)	5’- CACCCGCGAGTACAACCTTC -3’
β-actin: (R)	5’- CCCATACCCACCATCACACC -3’
Bcl-2: (F)	5’- TGTTGTTCAAAGGGATTCA -3’
Bcl-2: (R)	5’- GGCTGGGCACATTTACTGTT -3’
Bcl-XL: (F)	5’- GGCTGGGATACTTTTGTGGA -3’
Bcl-XL: (R)	5’- GGGAGGGTAGAGTGGATGGT -3’
Bax: (F)	5’- GGGGACGAACTGGACAGTAA -3’
Bax: (R)	5’- CAGTTGAAGTTGCCGTCAGA -3’
Bad: (F)	5’- CGGAGGATGAGTGACGAGTT -3’
Bad: (R)	5’- CCACCAGGACTGGAAGACTC -3’

Table S2. Criteria for OARSI Scoring

Osteoarthritic damage	Grade
Normal	0
Loss of Safranin-O without structural changes	0.5
Small fibrillations without loss of cartilage	1
Vertical clefts down to the layer immediately below the superficial layer and some loss of surface lamina	2
Verical clefts/erosion to the calcified cartilage extending to <25% of the articular surface	3
Vertical clefts/erosion to the calcified cartilage extending to 25-50% of the articular surface	4
Vertical clefts/erosion to the calcified	5

cartilage extending to 50-75% of the articular surface	
Vertical clefts/erosion to the calcified cartilage extending >75% of the articular surface	6
